# Passive Broad-Spectrum Influenza Immunoprophylaxis

**DOI:** 10.1155/2014/267594

**Published:** 2014-09-22

**Authors:** Cassandra M. Berry, William J. Penhale, Mark Y. Sangster

**Affiliations:** ^1^Molecular and Biomedical Sciences, School of Veterinary and Life Sciences, Murdoch University, Perth, WA 6150, Australia; ^2^Center for Vaccine Biology and Immunology, Department of Microbiology and Immunology, University of Rochester Medical Center, Rochester, NY 14642, USA

## Abstract

Influenza is a perennial problem affecting millions of people annually with the everpresent threat of devastating pandemics. Active prophylaxis by vaccination against influenza virus is currently the main countermeasure supplemented with antivirals. However, disadvantages of this strategy include the impact of antigenic drift, necessitating constant updating of vaccine strain composition, and emerging antiviral drug resistance. The development of other options for influenza prophylaxis, particularly with broad acting agents able to provide protection in the period between the onset of a pandemic and the development of a strain specific vaccine, is of great interest. Exploitation of broad-spectrum mediators could provide barricade protection in the early critical phase of influenza virus outbreaks. Passive immunity has the potential to provide immediate antiviral effects, inhibiting virus replication, reducing virus shedding, and thereby protecting vulnerable populations in the event of an impending influenza pandemic. Here, we review passive broad-spectrum influenza prophylaxis options with a focus on harnessing natural host defenses, including interferons and antibodies.

## 1. Introduction

Seasonal influenza causes serious disease burden, particularly in children and the elderly, with the need to develop annual vaccines based on predicted circulating strains due to antigenic drift of the virus. Furthermore, newly emerging novel influenza A viruses pose a significant threat of a pandemic with potentially devastating consequences [1]. Moreover, as pandemic strain vaccines require time for development and deployment, virus replication and spread are initially unchecked allowing the outbreak to gain momentum. Currently, vaccines and antivirals are used for control of influenza but emerging virus resistance to these measures presents limitations. Furthermore, as antiviral drugs could not be stockpiled in sufficient amounts for global supply, alternative control measures need to be urgently considered. A potential solution lies in the development of a universal vaccine based on conserved viral epitopes that induces cross-reactive antibodies that neutralize variant viruses from within a subtype and protects against heterologous viruses. Therefore, whilst existing vaccines are inadequate for cross protection and a universal vaccine may be difficult to achieve, it is both pertinent and timely to consider other possible broad-spectrum options, particularly as different virus subtypes not previously experienced by humans are emerging and have pandemic potential. In this review, we discuss broad-spectrum control options with emphasis on harnessing the power of natural immunity via neutralizing antibodies.

## 2. Influenza 

Effective passive broad-spectrum protection during the early phase of an epidemic could provide a barricade to virus exposure, especially during the interval between virus identification and active vaccine-induced immunity. As a consequence, early intervention with passive prophylaxis may revolutionize control options for influenza with potential impact on seasonal and pandemic influenza preparedness. Influenza A (H1N1 and H3N2) and influenza B viruses cause seasonal disease in the winter months of both hemispheres with 250,000–500,000 deaths each year [2]. Although the natural animal reservoir for influenza A viruses is aquatic shorebirds, high pathogenicity avian influenza (HPAI) viruses have recently emerged with high fatality rates in domestic poultry and species cross over into humans, with HA dual binding affinities for *α* 2,3-linked and *α* 2,6-linked sialic acid receptors, predominantly found in avian species and humans, respectively [3], allowing increased infectivity of avian viruses in the human respiratory tract. Mortality rates for HPAI H5N1 spreading throughout Eurasia and the newly emerged H7N9 virus in China are around 60% and 30%, respectively, for reported human cases of infection [4, 5]. At the present time, human-to-human transmission of these viruses is relatively rare. Confounding factors for avian influenza virus vaccine development include genetic engineering of the virus for better growth in eggs, poorer immunogenicity than seasonal influenza H1 and H3 subtypes in humans, and the need for use of higher doses and/or adjuvants to improve efficacy.

Four pandemics have occurred since the start of the 20th century, the most severe occurring in 1918 due to the Spanish flu pandemic with an estimated 50 million deaths [6]. Recently, novel H1N1 viruses emerged causing a pandemic in 2009 due to a natural reassortant swine influenza A virus, which was different to the H1N1 virus that had been cocirculating with H3N2 viruses since 1977 [7]. Reassortant viruses have been constructed in the laboratory to investigate their transmission and virulence, with one virus possessing the H5 HA gene derived from a HPAI H5N1 virus combined with the remaining seven gene segments from a 2009 H1N1 pandemic virus, displaying greater virulence than HPAI H5N1 with potential of increased mammalian transmission [8]. Thus we cannot be complacent with the continued threat of a pandemic by such new emerging pathogenic influenza viruses with increased mammalian transmission.

As influenza virus replication also impairs the mucociliary clearance, there is increased susceptibility to bacterial superinfections, which can often be fatal (reviewed in [9]). In addition, immune dysfunction in those prone to respiratory disease with excessive cytokine stimulation in the airways during influenza virus infection can rapidly progress to pneumonia with fatal acute lung injury [10–12]. Thus effective strategies to combat influenza pandemics of catastrophic proportions involve both the generation of broad-spectrum immunity and protection from immunopathology, thereby lowering the risk of severe illness-associated complications and reducing both morbidity and mortality.

## 3. Vaccines

Current influenza control requires effective vaccination. Updated vaccines with strain matching are developed on an annual basis requiring seed virus preparation generated by either reassortment or reverse genetics for propagation in eggs or cultured cells [13]. Recombinant DNA plasmid-based and virus-like particle vaccines are alternative options [14] to currently licensed inactivated and live attenuated vaccines (cold-adapted and temperature-sensitive viruses) [15] and recombinant proteins [16]. Heterosubtypic immunity is conferred by a variety of experimental vaccines including extracellular domain matrix (M2) protein [17] and formalin-inactivated whole virus [18]. Nonetheless, such contemporary vaccines would have narrow virus specificity, delayed availability, and restricted capacity to meet the global demand during a pandemic situation. Universal influenza vaccines that target invariant regions of the virus and induce effective broadly neutralizing immune responses could potentially provide more effective antiviral coverage but finding an appropriate immunogen is a major issue under intense investigation. A further limitation is efficacy as vaccine-induced B cell responses to conserved regions of the HA viral protein are generally low in frequency and generate poor antibody responses [19].

## 4. Antivirals

Neuraminidase inhibitors, although potent at inactivating virus replication, have been shown to display limited effectiveness when used beyond 48 hours after infection due to high viral titres and require regular dosing to those at risk of exposure during a virus outbreak. Other antivirals besides NA inhibitors (zanamivir and oseltamivir) that block the M2-ion channel (adamantanes) have been used; however prepandemic stockpiling of such antivirals by governments has been reconsidered as a poor strategy against emerging virus resistance (reviewed in [20]). Antiviral development has targeted specific oligosaccharide-comprised sites on enveloped viruses (cyanovirin) [21], viral RNA dependent RNA polymerase (favipiravir) [22], and their possible combination targeting different stages of the virus replication cycle in a synergistic therapeutic approach [23]. Innovative drugs may offer future clinical benefits including (DAS181) that mediates cleavage of sialic acid from host glycan receptors [24], *β*-defensins and antiviral peptides that prevent virion entry [25], TLR3 ligand (PIKA) promotion of DC maturation [26], TLR4 antagonists (eritoran) that block cytokine cascades [27], nucleic acid-based drug (PolyIC, CpG) activation of innate immunity [28], and siRNA that target conserved viral RNA sequences [29]. Furthermore, enveloped virus neutralizing compounds (polyphenols) found in pomegranate juice [30] and green tea [31] and even scorpion venom peptide [32] hold promise in their strain diversity-independence of antiviral activity.

## 5. Innate Interferons

**Figure 1:**
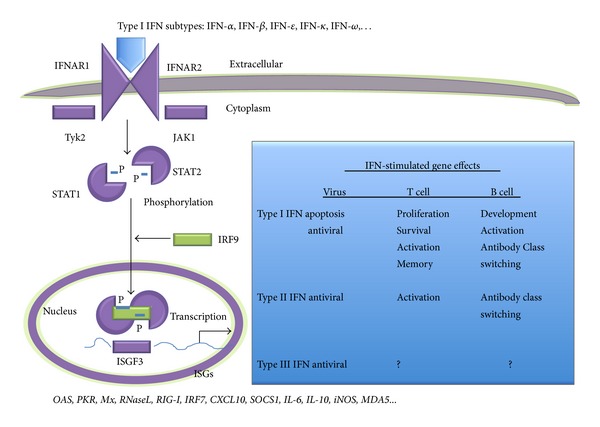
Signal transduction pathway for type I IFNs and a summary of key ISG effects on the virus and mucosal immune response during influenza. Type II IFN (IFN-*γ*) binds IFNGR1 and IFNGR2 heterodimer receptor on the cell surface, which leads to activation of JAK1 and JAK2, STAT dimerization, and phosphorylation with transcriptional activation of ISGs with the GAS element. Type III IFNs (IFN-*λ*1, -*λ*2, and -*λ*3) signal via ligand binding to IFNLR1 and IL-10R2 subunits of the cell surface receptor, activation of JAK1 and Tyk2 with similar downstream signaling pathways as the type I IFNs. ISGs can be either discrete or common sets for the different IFN families. Biological properties of type I, type II, and type III IFNs as identified in T and B cells [33–39] do not represent an exhaustive list.

Innate IFNs are potent pleiotropic cytokines that can stimulate cells to induce an antiviral state through signal transduction pathways activating transcription of specific gene subsets. The IFN molecules comprise multiple subtype proteins belonging to the types I, II, and III families and play important roles in early host defense against virus infections through their diverse antiviral and immunomodulatory properties (Figure 1). IFN-α/*β* are the best characterized of the type I IFN family, which is comprised of IFN-α (1–14 subtypes), beta, tau, epsilon, omega, and many more. However, influenza viruses can evade innate immunity in part through the nonstructural- (NS-) 1 protein, targeting interferon regulatory factor- (IRF-) 3, blocking IFN promoter activity and antagonizing endogenous IFN pathways [40].

IFNs [41] or IFN-inducible proteins (viperin) [42] provide broad-spectrum protection against influenza A viruses. The IFNs have been shown to activate transcription of about 2,000 IFN-stimulated genes (ISG) [33] with heterogeneous antiviral activity in mammalian cells [34]. Major antiviral states are induced by* RNaseL*, inhibiting viral nucleic acid replication [35],* 2-5OAS*, blocking virus protein translation [36], and* Mx*, abrogating the formation of viral ribonucleoproteins [37]. IFNs can also affect immune cell proliferation, differentiation, maturation, migration, and survival. IFNs orchestrate the downstream adaptive immune responses by activating B cells to switch antibody class secretion and by stimulating T cell proliferation and survival to sustain T cell memory. Although each protein of the type I IFN family binds a single type of cell surface receptor (IFNAR), the binding affinities and downstream signaling pathways differ resulting in activation of ISG subsets influenced by virus and cell type [38].

IFNs have been used for clinical therapy of a variety of infections and diseases. Although IFN therapy predominantly utilizes IFN-α2, other subtypes may be more or less effective, depending on the virus and cell type [39]. Differential efficacies have been found for individual IFN subtypes in mouse models of influenza, with IFN-α5 and IFN-α6 subtypes being more effective than IFN-α1 in reducing lung H1N1 influenza virus titers [43]. The IFN-*β* subtype has also been shown to protect against H1N1, driving a pulmonary Th1 type response beyond day 8, which normally switches to Th2 with eosinophilia/neutrophilia during natural infection [44].

IFN delivery has often been via bolus injections, which can have adverse side effects including severe influenza-like illness with fatigue and depression [45]. Intranasal instillations of IFN to the respiratory mucosa can protect susceptible type II alveolar epithelial cells from influenza virus infection but is associated with various efficacies in preclinical trials [46]. Questions that still need to be adequately addressed are the timing, route, and dosage of IFN regimens for effective cover as a protective measure against influenza. Importantly, exogenous IFN cannot be immunogenic; otherwise neutralizing antibody responses develop, rendering treatment ineffective [47]. In an attempt to overcome adverse side effects with current modes of IFN-α treatment, a recent clinical trial investigating administration of low-dose oral IFN-α prophylaxis was undertaken but was deemed ineffective in protection against acute respiratory illness during the 2009 influenza pandemic [48].

More recently, the type III IFN-lambda family was discovered (*λ*-1-3) and found to induce similar antiviral effects to the type I IFNs except for their cell-restriction and signaling mainly in epithelial cells and hepatocytes [49, 50]. Although these IL-10-like cytokines bind receptors distinct to the type I IFNs, most cells in the body can produce both IFN types, which are induced by TLR activation and internal sensors such as RIG-I and MDA5. Both IFN-α/*β* and IFN-*λ* have been shown to be important for control of influenza in experimental animal models utilizing double receptor knockout mice [51]. Paradoxically, IFNs may either reduce or enhance inflammatory conditions [52], and as such their repeated applications to the respiratory tract need to be addressed in clinical trials. Importantly, unregulated IFN protein levels can exacerbate lung inflammation and immunopathology [53]. Regulation of IFN pathways is complex, mediated by receptor density, binding affinity, STAT phosphorylation and multimerization, ISG transcription, and regulatory feedback loops. Unless an improved safety regime is utilized, clinical studies using type I and type III IFN appear to show limited potential for influenza prophylaxis and are unsuitable for long-term protection of the respiratory mucosa. Further limitations of exogenous IFN production capacity and species-specificity of IFN signaling may be disadvantages in pandemic control.

## 6. Neutralizing Antibodies

**Figure 2:**
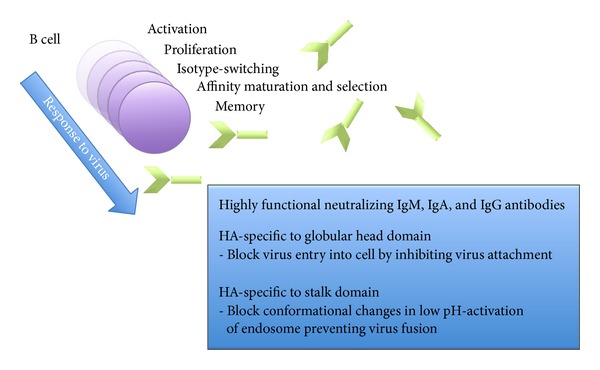
Activities of neutralizing antibodies to influenza virus induced by B cell stimulation. Influenza virus stimulates B cell responses through a sequential process of activation, proliferation, isotype-switching, affinity maturation and selection of antibodies, and memory. The major activities of neutralizing antibody types to viral HA are shown in the inset box [13]. HA-specific antibodies are highly functional and prevent virus infection resulting in less cells being infected* in vivo*.

Stimulation of polyclonal B cells can generate circulating antibody pools with multiple epitope reactivity induced by vaccination or natural infection with influenza virus. Antibodies blocking the binding of viral HA proteins to cell surface receptors directly neutralize the virus and prevent virus entry. Once the virus infection cycle has been abrogated by such humoral immune responses, the spread of virus to neighboring cells is inhibited, virus shedding is reduced, and the infection cleared through a combined antibody and effector T cell response [54], extensively reviewed elsewhere [55]. Importantly, the production of highly functional neutralizing antibodies against HA epitopes of influenza viruses, even at nominal levels, is a relative correlate of protection [56] (Figure 2). Cognate signals and secreted factors from activated CD4^+^ T helper cells also activate B cells to secrete virus-specific antibody. Extrafollicular antibody-secreting cells that mainly produce early IgM are regulated by type I IFN and IL-12 cytokines, whereas inherent innate host factors influence mature B cells through a process of class switching to produce IgG during the course of influenza virus infection [57]. Memory B cells and antibody-secreting cells formed in germinal centers can produce virus-specific antibody in a recall response to secondary virus infection [58]. Trivalent inactivated and live attenuated vaccines predominantly induce humoral responses with neutralizing antibodies directed against the HA globular head domain, which block specific virus binding thus inhibiting virus entry. However, such antibody specificities can be limited in efficacy against variable viral sequences due to the high mutation rate in the HA head domain.

## 7. Passive Antibody-Based Immunoprophylaxis

Currently, there is renewed interest in passive immunity mediated by neutralizing antibodies for protection against influenza [59–62]. Convalescent sera from survivors of the H1N1 Spanish Flu pandemic were reported to reduce mortality rates [63] and convalescent plasma treatment was deemed effective for H5N1-infected individuals in China [64] and for the pandemic H1N1 in Hong Kong [65]. It is noteworthy that even a relatively low titre of anti-HA antibody in convalescent plasma (HAI titer 128) protected 100% ferrets from a fatal HPAI H5N1 virus infection, when passively transferred 24 hours before virus challenge [66].

Intranasal delivery of bovine IgG obtained from colostrum was found to provide passive prophylaxis for influenza in mice, when both specificity of antibodies and challenge virus were strain-matched [67]. Broader efficacy of antibodies with neutralizing capacity may be achievable with a mixture of polyclonal antibody specificities raised against multivalent HA vaccines. Alternatively, broadly neutralizing antibodies to conserved regions of the HA capable of neutralizing different strains within a subtype and group or between groups and types of influenza viruses may be effective as a prophylactic interim therapy. In addition to antihead HA antibodies, some antistalk HA antibodies have been shown to block fusion [68] and promote antibody-dependent cellular cytotoxicity of infected cells with broad neutralizing capacity [69]. As routine haemagglutination inhibition assays do not detect antistalk antibodies, the discovery and potential of HA-stalk-specific antibodies largely remained unknown for some time [70]. An advantage over anti-HA head antibodies that are prone to escape mutations is that certain antistalk antibodies are less susceptible to mutations, as the stalk epitopes in the membrane-proximal region are critical for virus function [71]. Rather than attempt to directly induce such broadly neutralizing antibodies in humans by vaccination, which is limited to finding an appropriate immunogen with approved adjuvants, such antibodies may be more practically and effectively generated in naïve donor animals with optimal vaccination protocols for prophylaxis in humans. However, knowledge of the protective coverage, dosage, and route of administration of such antibodies needs to be gained from further investigations.

Monoclonal neutralizing antibodies cross protective against H1, H2, H5, H6, and H9 influenza virus strains were recovered from human IgM^+^ memory B cells [72]. Human monoclonal antibodies have also been identified with broad-spectrum prophylactic efficacy against HPAI H5N1 influenza viruses in mice and ferrets [73, 74]. Memory from past exposure to H1N1 influenza virus strains was also apparent in heterovariant immunity of individuals over 30 years of age rather than those younger during the H1N1 pandemic in 2009 [75]. Various monoclonal IgG antibodies against HA epitopes have been investigated for broad-spectrum prophylaxis in mice and their efficacy compared to the antivirals, amantadine, oseltamivir, and zanamivir [76]. Use of humanized and chimeric virus-specific monoclonal antibodies [77] and Fab fragments has been explored in recent years to circumvent immunogenicity issues and address suitability of such antibodies for broad-spectrum passive prophylaxis; however their development and supply may not be practical and cost effective in pandemic control of influenza. Furthermore, the optimal dose and mode of delivery of a single monoclonal antibody preparation to the stalk domain are issues, with much higher titres needed for parenteral delivery as opposed to intranasal delivery [78].

Polyclonal antibodies with high affinity targeting multiple epitopes of the viral HA protein are under substantial investigation for influenza control. IgG antibodies have been shown to play a major role in protection of ferrets from homologous and heterologous H5N1 influenza viruses [79]. As mentioned before, select regions of the head and stalk domains of the HA molecule are highly conserved and can induce cross protective antibody responses, albeit suboptimal. As the HA head is immunodominant, stalk antigens have relatively low immunogenicity and do not stimulate large amounts of highly reactive antibodies with classical vaccine regimes. However, broadly neutralizing high affinity antibodies could potentially be prepared in animals with immune manipulation using modified vaccine regimes and better adjuvants than those licensed for human use [80]. Vaccination of naïve animals to generate heterologous polyclonal antibodies has benefits in targeting responses to potential key epitopes, since preexisting memory B cells with specificities for other immunodominant epitopes are not present to outcompete the naïve B cells. As an example, prime-boost vaccination strategies have been found to increase neutralizing antibody titers 30- to 50-fold and reduce lung virus loads by 2 logs [81–83], expanding the number and diversity of activated HA-specific CD4^+^ T cell clones.

Although universal vaccination in humans is the ultimate goal [84, 85], broadly neutralizing antibodies produced in a large animal donor with strategic vaccination regimes would be useful as an interim therapy for influenza. Heterologous antibodies could be passively administered by the intranasal route to provide immediate protection of the respiratory tract mucosa. Intranasal delivery of broadly neutralizing antibodies has been investigated in mice affording protection [86–89] and warrants further studies in preclinical models. Vaccination of large ruminant animals, such as dairy cows, is currently being considered and has the potential to supply a bulk source of milk-derived neutralizing antibodies for clinical trials. Clearly, many unanswered questions regarding efficacy of routes of administration, dosage, timing, and immune memory to reexposure need to be addressed in future investigations of antistalk HA antibody-based prophylaxis for influenza.

## 8. Pandemic Control

**Figure 3:**
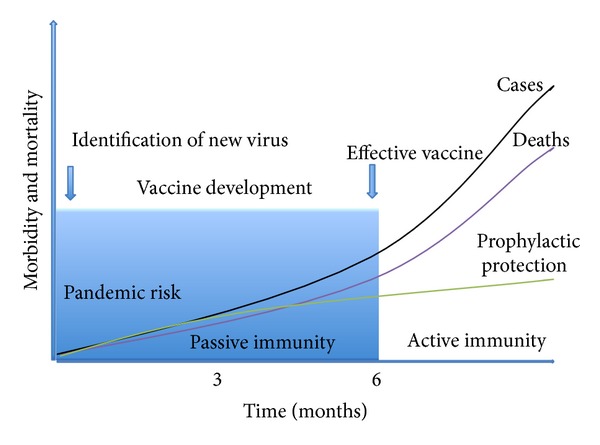
Modeling of the impact of prophylaxis on mortality curves during pandemic influenza. Prophylaxis could protect against both morbidity and mortality rates by lowering the number of cases. Effective passive immunity is important to provide coverage during the first wave or six-month risk period of the pandemic whilst an effective vaccine is developed for the identified virus to induce active immunity.

Despite modern advances in vaccine technology with the availability of antivirals and antibiotics and improved global surveillance of influenza virus, a contemporary pandemic may have a worse case scenario than that which occurred in 1918 [90]. Compounding these factors is civil unrest and the existence of poor medical infrastructure in overcrowded developing countries, likely to be the epicenter of newly emerging influenza virus outbreaks. Clearly, intervention needs to be early, effective, and robust in the effort to curtail morbidity and mortality (Figure 3). Antibody-based passive immunity is a powerful prophylactic strategy for influenza pandemic control, reducing the risk of virus spread in populations. Ideally, an antibody-based therapy could be self-administered, via either an inhaler or intranasal spray for protective coverage of the airways during the first wave of a pandemic. The dairy industry infrastructure in some countries (e.g., Australia) provides an opportunity for bulk antibody production in large ruminants. One may speculate that if a single 50 mg dose of potent broadly neutralizing antibodies could be effective over a 7-day duration, coverage for 3 months would require 12 doses that possibly could be distributed to families and self-administered. Assuming average annual milk production of 6,000 L/cow, it is feasible that approximately 6 kg of immunoglobulin (10,000 doses) could be produced per cow. In addition to those susceptible and at high risk of exposure, an immunoprophylaxis option would also be particularly important for those who have been previously vaccinated with seasonal influenza and may be unresponsive to pandemic influenza vaccination due to a transient antiviral state, recently termed innate imprinting [91]. Monoclonal antibody-based immunotherapy with broadly neutralizing capabilities and universal anti-influenza vaccines show promise for influenza prophylaxis, affording protection of the respiratory tract mucosa (reviewed in [92]). Interest in polyclonal antibody-based immunotherapy has recently been reignited with a study showing neutralizing efficacy of antistalk antibodies with new modes of action in a ferret model [93].

The prospects of producing bulk supplies of antibodies in large production animals present a strategic approach, likely to generate a more robust antibody response in the donor animal, which can be passively transferred, than that directly induced by natural virus infection or currently licensed vaccines and adjuvants for use in humans. As an alternative to animal-based production of antibodies, the expression of therapeutic antibodies in tobacco plants has also recently been of interest for treatment of the current Ebola virus outbreak in West Africa [94], although scaleup for adequate supplies may be a limitation. In a preclinical model, we have investigated intranasal delivery of ruminant polyclonal antibodies to protect the respiratory mucosa of mice against infection with H1N1 influenza virus [95]. We found that exogenous IgG, derived from H1N1-immunized sheep, as a model ruminant, protected mice upon virus challenge. Protection was Fc-independent, was effective up to 3 days before virus exposure, and largely prevented infection in the lungs and preserved normal lung architecture. A benefit of intranasal administration is the direct noninvasive delivery of antibody to the lumen of the respiratory mucosa, whereas in a natural influenza virus infection, the presence of IgG in the airways is derived from circulating antibody by transudation with IgM and IgA transported across the epithelium to the lumen. Investigations of antistalk antibody isotypes and subclasses generated in immunized donor animals are required to increase knowledge of optimal efficacy and cross protection against clades spanning H1, H3, H7, and H9 virus types.

## 9. Conclusion

Antiviral prevention strategies for control of newly emerging or reemerged influenza virus strains of pandemic potential are of utmost importance. Whilst intense efforts are being made to meet the urgent need for a universal vaccine eliciting long-term immunity, passive immunoprophylaxis would have benefits of immediate short-term protection, especially of benefit to the elderly and very young ones, who have poorer antibody responses to vaccination. Control of influenza with antiviral drugs lowers the risk of complications [96] but drug resistance can develop, with adamantanes no longer recommended for use [20]. Furthermore, cost-effective management in low resource settings is problematic in many endemic regions, especially in South East Asia. Broad-spectrum immunoprophylaxis holds future promise as an effective control measure for influenza. Preparation of broadly neutralizing antibodies in naïve animal donors is potentially inexpensive and independent of host immune memory to influenza viruses, where dominant responses from HA-specific memory B cells are often induced by vaccination or natural infection, a phenomenon first described as original antigenic sin [97, 98]. In conclusion, serious concerns may be mitigated by passive immunoprophylaxis using broadly neutralizing antibodies that provide universal coverage against multiple influenza viruses, providing an interim control option for pandemic influenza.
